# From algorithm to alarm: a narrative review on insulin delivery innovation

**DOI:** 10.1097/MS9.0000000000005307

**Published:** 2026-07-17

**Authors:** Noor Ul Ain Saleem, Faraz Arshad, Maria Qadri, Muhammad Saqlain Mushtaq, Mishaim Khan, Pakeeza Shakoor, Tirath Patel, Anaya Noor, Nana Sardarova, Nikhilesh Anand, Carolyn K. Edmondson, Adekunle E. Omole

**Affiliations:** a Department of Medicine, FMH College of Medicine and Dentistry, Lahore, Pakistan; b Department of Medicine, Shaikh Zayed Hospital, Lahore, Pakistan; c Department of Medicine, Jinnah Sindh Medical University, Karachi, Pakistan; d Department of Medicine, Shifa College of Pharmaceutical Sciences, Shifa Tameer-e-Millat University, Islamabad, Pakistan; e Department of Medicine, Karachi Medical and Dental college, Karachi, Pakistan; f Department of Medicine, Trinity Medical Sciences University School of Medicine, Saint Vincent and the Grenadines, Kingstown, Saint Vincent and the Grenadines; g Department of Medicine, Dow Medical College, Karachi, Sindh, Pakistan; h Department of Internal Medicine, Henry Ford Hospital, Detroit, USA; i Department of Medical Education, University of Texas Rio Grande Valley, Edinburg, TX, USA; j Clinical Medicine, American University of Antigua, St. John, Antigua and Barbuda; k School of Engineering Medicine, Texas A&M University, Houston, USA

**Keywords:** artificial pancreas, closed-loop insulin delivery, insulin delivery systems, management of diabetes mellitus, sensor-augmented insulin delivery

## Abstract

**Background and objective:**

The global rise in the incidence of diabetes mellitus necessitates innovative management strategies to mitigate the severity of the disease. The mainstay of diabetes treatment is still insulin therapy, although there are drawbacks, such as the need to maintain ideal glycemic control while lowering the risk of hypoglycemia and user fatigue. The development, clinical usefulness, and constraints of insulin delivery systems are examined in this narrative review, with particular attention to sensor-augmented and closed-loop technologies.

**Methods:**

A thorough electronic database search was conducted to obtain pertinent data on major insulin delivery devices, including sensor-augmented and closed-loop systems. We studied advancements in insulin delivery systems over time and their impact on the management of diabetes mellitus. We thoroughly examined both qualitative and quantitative data to summarize our conclusions.

**Key content and findings:**

Closed-loop insulin delivery systems, which combine insulin pumps, advanced control algorithms, and continuous glucose monitoring, are the next step forward in the treatment of diabetes. These systems autonomously regulate blood glucose levels, reducing the need for user intervention. Closed-loop devices outperform sensor-augmented pump (SAP) treatment in reducing hypoglycemia, extending the time-in-range, and lowering self-management responsibilities. In order to provide real-time control, closed-loop systems dynamically modify insulin delivery in response to glucose variations by using algorithms powered by artificial intelligence. Because SAP therapy depends more on user input and may not be as successful in providing consistent glycemic control, this capability helps overcome its drawbacks. Further improving overall safety and efficacy, the closed-loop technique decreases blood glucose variability.

**Conclusion:**

The two primary developments in diabetes care are sensor-augmented and closed-loop insulin devices, which offer better glucose control and lower hypoglycemia rates compared to traditional therapies. Closed-loop insulin delivery systems seem to be a more clinically successful option for controlling diabetes mellitus than SAP therapy.

## Introduction

Diabetes mellitus (DM) is a group of metabolic disorders characterized by a chronic hyperglycemic condition resulting from defects in insulin secretion, insulin action, or both^[^1^]^. The global prevalence of diabetes in 2019 was estimated at 9.3% (463 million people), rising to 10.2% (578 million) by 2030. Just under half a billion people are living with diabetes worldwide, and the number is projected to increase by 25% in 2030 and 51% in 2045^[^2^]^. DM has several categories, including type 1, type 2, maturity-onset diabetes of the young, gestational diabetes, neonatal diabetes, and secondary causes due to endocrinopathies and steroid use. The main subtypes of DM are Type 1 diabetes mellitus (T1DM) and Type 2 diabetes mellitus (T2DM)^[^3^]^. In the management of all diabetic patients, the primary therapeutic goal is to achieve reasonable glycemic control (GC) to prevent macrovascular and microvascular complications^[^4^]^.

**Table 1:** Comparative analysis between closed-loop insulin delivery systems and sensor-augmented insulin delivery systems.

Aspects	Closed-loop insulin delivery systems	Sensor-augmented insulin delivery systems
Key components	Continuous glucose sensor Insulin pump Control algorithm	Continuous glucose sensor Insulin pump
Glucose monitoring methods	Interstitial glucose sensing Subcutaneous glucose sensor Microdialysis, Amperometric enzyme electrodes	Interstitial glucose sensing Subcutaneous glucose sensor
Insulin delivery method	Subcutaneous insulin pump	Subcutaneous insulin pump
Control algorithm	Model predictive control (MPC) Proportional-integral-derivative control (PID)	MPC PID
Artificial pancreas types	Single-hormone systems (insulin delivery); dual-hormone systems (insulin and glucagon delivery)	N/A
Effectiveness in glucose control	Enhances glucose control in outpatient settings Improves night time glycemic control, and reduces the likelihood of hypoglycemia	Superior to traditional continuous subcutaneous insulin infusion (CSII) Improves HbA1c levels, enhances safety with automatic suspension of insulin delivery during hypoglycemic events
Safety and efficacy	Safe and effective for various age groups Reduced hypoglycemia compared to conventional pump therapy Viable and safe for young individuals with type 1 diabetes	Safe and effective for various age groups Increases time in range Reduces overnight hypoglycemia Better glucose control compared to traditional subcutaneous insulin therapy

**Table 2:** Closed-loop and sensor-augmented insulin delivery systems: mechanisms, clinical outcomes, and effectiveness.

	Closed-loop insulin delivery system	Sensor-augmented insulin delivery system
Mechanism	Integrates three key components: Continuous glucose sensor, insulin pump, and control algorithm. The system automates insulin delivery based on real-time glucose data, adjusting insulin doses accordingly.	Combines a continuous glucose sensor with an insulin pump. The sensor provides real-time glucose readings, which are used to adjust insulin delivery. This integration allows for more precise insulin dosing in response to changing glucose levels.
Clinical outcomes	Enhanced time in range (3.9–10.0 mmol/L) Reduced hypoglycemia (<3.9 mmol/L) Improved overall glycemic management Reduced hypoglycemia time, leading to fewer hypoglycemia episodes	Improved time in range (3.9–10.0 mmol/L) Reduced hypoglycemia (<3.9 mmol/L) Enhanced safety with automatic suspension of insulin delivery during hypoglycemic events, improved overall glycemic management, reduced risk of hypoglycemia episodes
Effectiveness in glucose control	Consistently enhances glucose control by optimizing insulin delivery based on real-time glucose data. This leads to improved time in range, reduced hypoglycemia episodes, and better overall glycemic management.	Improves glucose control by providing real-time glucose readings and adjusting insulin delivery accordingly. This helps reduce hypoglycemia episodes and enhances safety by automatically suspending insulin delivery during potential or actual hypoglycemic events.

GC in T2DM patients can be evaluated using three parameters: glycosylated hemoglobin (HbA1c), fasting blood glucose, and postprandial glucose^[^5^]^. Intensive blood glucose control with insulin substantially decreases the risk of microvascular complications but not macrovascular disease in patients with type 2 diabetes^[^6^]^. Insulin therapy is a cornerstone in the management of diabetes. It involves administering exogenous insulin to regulate blood glucose levels. Intensification of insulin therapy has become an essential treatment method, but an increased risk of hypoglycemia has hindered it. Severe hypoglycemia can be life-threatening and negatively affect daily life and work. Despite its effectiveness, insulin therapy comes with several challenges^[^7^]^. These challenges include clinician-related factors (such as staying informed and optimizing adherence), patient-related concerns (including fear and education), self-management challenges (lifestyle awareness and insulin delivery methods), and healthcare system issues (such as cost control). Regular monitoring, adjusting insulin doses, and lifestyle modifications are required for better results^[^8^]^.

Closed-loop and sensor-augmented insulin delivery systems are technological advancements in diabetes management. Closed-loop (artificial pancreas) systems for automated insulin delivery have been likened to the holy grail of diabetes management. Progress toward an entirely closed-loop system has been accelerating since the mid-2000s, with the development and commercialization of numerous glucose-sensing and insulin-delivery systems of increasing sophistication^[^9^]^. The past decade has seen rapid advances in the development and uptake of closed-loop systems, with the hybrid closed-loop system transitioning from research to commercial availability. Although the ultimate artificial pancreas, an entirely closed-loop system, has not yet been realized in clinical practice, the success of closed-loop system development thus far and the timeline it has achieved are promising^[^10,11^]^. In this review, we compare the current status of closed-loop insulin delivery systems and sensor-augmented systems, focusing on their clinical applications and evaluations. We outline future goals and contrast the benefits and limitations of closed-loop therapy and sensor-augmented pump (SAP) therapy.

This manuscript is made compliant with the TITAN checklist to ensure transparency in the reporting of artificial intelligence (AI)^[^12^]^.

## Methods

### Literature search strategy

We extensively searched several electronic databases, namely PubMed, MEDLINE, Scopus, Embase, and Google Scholar. These databases contain medical literature, peer-reviewed articles, reviews, and historical documents. We utilized a combination of medical subject headings (MeSH terms) and keywords to optimize our search strategy. The relevant terms we focused on included “closed-loop insulin delivery,” “sensor-augmented insulin delivery,” “artificial pancreas,” “diabetes management,” “insulin delivery,” “continuous glucose monitoring,” and “algorithm.” Boolean operators and additional search terms were also used to refine the results and address specific subtopics.

### Study selection criteria

To ensure our study includes the most relevant and up-to-date findings, we primarily focused on studies published in English between January 2005 and June 2025, which provided the foundational knowledge required for our review. We identified 688 relevant articles, of which 51 were included in the final narrative synthesis. Eligible studies comprised randomized controlled trials, observational studies, systematic reviews, meta-analyses, and significant narrative reviews that reported clinical outcomes, including GC, hypoglycemia incidence, time-in-range (TIR), patient satisfaction, or safety. Studies that were restricted to *in vitro* tests or animal models, that only addressed non-insulin treatments such as oral hypoglycemics, or that were case reports, editorials, or conference abstracts lacking peer-reviewed data were all disqualified. This review employs a narrative method, facilitating the qualitative synthesis of both historical and contemporary evidence. Information gathered from selected sources was organized both chronologically and thematically to construct a coherent narrative outlining the new innovative developments and their impact on diabetes management. The findings from the included studies were summarized using narrative means. Common themes and patterns observed across studies were identified and synthesized.

HIGHLIGHTS

- Continuous glucose monitoring (CGM), insulin pumps, and advanced algorithms that automatically modify insulin supply to drastically lower hypoglycemia and improve time-in-range are all combined in closed-loop insulin delivery systems (artificial pancreas) to provide superior glycemic management.
- Although sensor-augmented pump therapy still necessitates a large amount of user input, it enhances conventional insulin pump therapy by including CGM, enabling improved glucose management and safety features, such as automatic insulin suspension during hypoglycemia.
- Closed-loop systems are now more effective, efficient, and user-friendly across various age groups, particularly young children and elderly people, because of technological breakthroughs like artificial intelligence and machine learning integration. This has improved the quality of life and decreased the burden of diabetes.
- Racial and socioeconomic differences restrict the equitable benefits of advancements in insulin delivery, and disparities in access to this cutting-edge technology continue to be a significant global concern.
- Future directions center on developing completely automated systems with dual-hormone delivery, faster-acting insulin, and enhanced sensor accuracy; however, existing constraints such as cost, calibration requirements, and psychological hurdles continue to impede general use.

## Discussion

### Closed-loop insulin delivery system

Automated closed-loop insulin delivery, often dubbed the “artificial pancreas,” integrates three key components: a continuous glucose sensor, an insulin pump, and a control algorithm^[^13^]^.

#### Benefits and impact

The use of continuous glucose monitoring (CGM) devices alone has been shown to improve adult glucose homeostasis and reduce hypoglycemia episodes in some populations of individuals with type 1 diabetes who are under good management. Today’s closed-loop systems use subcutaneous insulin pumps, interstitial glucose monitoring, and extremely complex algorithms. This automated method of delivering insulin has greatly reduced human error in GC and eased the everyday strain of self-management activities for diabetics^[^13^]^.

### Glucose sensing technologies

#### Non-invasive sensors

Both minimally invasive and non-invasive glucose sensors are available to measure glucose levels in interstitial fluid. Optical glucose sensors are frequently employed for non-invasive techniques. These sensors pass a light beam through the skin and examine any changes in the characteristics of the reflected light. However, because sample extraction and analysis are required, it takes about 20 minutes to receive measurement results. Furthermore, the skin may become irritated by the current used during this procedure^[^14^]^.

#### Minimally invasive sensors

Amperometric enzyme electrodes and microdialysis are two minimally invasive techniques for glucose monitoring^[^14^]^. A semi-permeable dialysis fiber is placed into subcutaneous tissue and perfused with glucose-free fluid in microdialysis, such as Menarini’s Glucoday® system. After diffusing into the perfusate, glucose is continually delivered to an external glucose sensor for ongoing monitoring. Time lag is a disadvantage due to tubing length and perfusion flow rate, even when there is no discernible signal drift. As an alternative, subcutaneously placed electrodes coated with enzymes can identify changes in current that correspond to glucose levels. However, frequent recalibration is necessary to address signal drift caused by tissue response and glucose transport close to the electrode^[^15^]^.

### Insulin delivery mechanisms

A portable electromechanical pump is used in the conventional continuous subcutaneous insulin infusion (CSII) technique to mimic the insulin administration patterns of people without diabetes. It delivers insulin at preset rates, usually with a steady baseline rate augmented by patient-initiated increases at mealtimes^[^16^]^.

### Control algorithms

#### Model predictive control

A technique that links insulin administration and food consumption to variations in blood sugar levels is called model predictive control (MPC). It may be a complex model that accounts for the body’s natural glucose regulation or a simpler “black box” model that uses pattern recognition techniques to learn about insulin and glucose trends. Mathematical models of how the body regulates blood sugar can be used in both methods. Minimizing the difference between the model’s expected and desired glucose levels over time yields the insulin rate^[^17^]^.

#### Proportional-integral-derivative control

The insulin delivery rate is controlled by the proportional-integral-derivative (PID) controller, also referred to as PID control, which examines glucose changes from three perspectives: the distance from the target glucose level (proportional), the area difference between the actual and target glucose levels (integral), and the rate of change in measured glucose levels (derivative)^[^18^]^.

### Types of closed-loop systems

#### The artificial pancreas

The artificial pancreas, a closed-loop insulin administration system, is created by combining insulin pumps and CGM monitors. In this case, a sensor sends interstitial glucose data to a controller, which communicates with the user and executes a control algorithm. Real-time insulin delivery through a pump is adjusted by the control algorithm. Components communicate with each other wirelessly. There are two types of artificial pancreas: single-hormone systems that release only insulin, and dual-hormone systems that release both insulin and glucagon. The dual-hormone approach may allow for more assertive insulin administration, despite its complexity. The results of the study show that, in comparison with traditional pump therapy, artificial pancreas systems consistently improve glucose management in outpatient settings^[^19^]^.

#### Clinical evidence and real-world application

- **Safety and feasibility**: For young people with type 1 diabetes, research suggests that unsupervised day-and-night hybrid closed-loop insulin delivery at home is feasible and safe. In teenagers with poorly managed type 1 diabetes, this indicates that closed-loop insulin delivery, rather than sensor-augmented insulin pump therapy, may improve glucose control without increasing the risk of hypoglycemia^[^20^]^.
- **Efficacy**: Compared to traditional pump therapy, artificial pancreas devices drastically cut down on hypoglycemia time, reducing it by 2.45%, or roughly 35 minutes less per day. Both 24-hour and overnight investigations consistently show this decrease, with the overnight decrease being more noticeable. Blood glucose levels in type 2 diabetes can also be effectively managed using a fully closed-loop insulin administration device^[^21^]^.

### Sensor-augmented insulin delivery systems

The broad availability of blood glucose self-monitoring made CSII easier to use. This guaranteed efficient management of the infusion system (which includes the pump, reservoir, and infusion set) and enabled insulin modifications based on the patient’s immediate needs^[^22^]^. SAP therapy, which combines CSII with CGM, is more effective than regular CSII in studies such as the REAL Trend study and significantly lowers HbA1c levels. By immediately stopping insulin delivery during possible or actual hypoglycemic episodes, the Paradigm® VEO system improves safety by lowering the risk of hypoglycemia and diabetic consequences^[^23^]^. However, in conclusion, closed-loop therapy proved safe and effective for older adults with long-duration type 1 diabetes, showing increased time in range and reduced overnight hypoglycemia compared to SAP therapy.

### Global and real-world perspectives

According to Wilson, Leah *et al*, by raising glucose TIR (3.9–10.0 mmol/L) and lowering hypoglycemia (<3.9 mmol/L) in individuals with type 1 diabetes, commercial automated insulin delivery therapy might enhance glucose outcomes^[^24^]^. Overnight, closed-loop insulin delivery improved the percentage of time within, above, and below the CGM goal range. This was its most significant advantage. The fourfold reduction in time spent below all hypoglycemic thresholds assessed in the study with a closed-loop system ^[^25^]^ was significant from a therapeutic standpoint for this older group. In adults with type 1 diabetes, a systematic review and meta-analysis compared the safety and effectiveness of SAPs versus closed-loop insulin administration. According to the investigation, closed-loop systems performed better overall in controlling blood sugar levels and had fewer hypoglycemia episodes^[^26^]^. Patients with type 1 diabetes were randomized 2:1 to receive therapy with a closed-loop device (closed-loop group) or an SAP (control group) in a 6-month randomized multicenter experiment. The glycemic advantages of closed-loop management were observed throughout the trial’s first month and the full 6 months ^[^27^]^. Ware *et al* discovered that children using the CamAPS FX closed-loop system had an 8.7 percentage point higher percentage of time that their glucose level was in the target range than children using an SAP (without automation) in another randomized crossover trial with 74 children ranging in age from 2.3 to 7.9 years (mean, 5.6 years)^[^28^]^. An automated, closed-loop insulin delivery device produced noticeably better glucose control than traditional subcutaneous insulin therapy in type 2 diabetic inpatients receiving noncritical care without increasing the risk of hypoglycemia^[^29^]^. The closed-loop technology did not appear to cause any unexpected safety issues for patients in our trial. In both groups, the rates of hypoglycemia (as determined by CGM) and clinically severe hypoglycemia were modest and comparable. In patients using insulin pumps, pump infusion-set failure frequently results in hyperglycemia with or without ketosis. More instances of this type of failure were observed in the closed-loop group than in the standard care group. Compared to standard-care insulin delivery using a continuous glucose monitor, the hybrid closed-loop device produced a higher proportion of glucose levels in the target range over a more extended period in this 13-week trial involving children with type 1 diabetes who were 2–6 years old^[^30^]^. When compared to sensor-augmented insulin pump therapy, the 12-week use of a hybrid closed-loop insulin delivery system during the day improved overall glucose control and decreased the risk of hypoglycemia in individuals with suboptimally controlled type 1 diabetes, including adults, adolescents, and children^[^31^]^.

### Technological innovation and advancement

Closed-loop systems, also called artificial pancreas systems, are state-of-the-art devices that automate the supply of insulin to people with diabetes. These devices continuously check glucose levels and modify insulin dosages in real time to keep blood sugar levels steady. Their goal is to lessen the strain of routine insulin injections and blood glucose monitoring, particularly in young children^[^32^]^. These systems have evolved into fully automated versions requiring minimal user input, improving diabetes management safety and efficacy. Closed-loop devices offer a more practical and effective means of controlling blood sugar levels, a hopeful development in treating diabetes. When managing type 1 diabetes, closed-loop insulin delivery devices have proven more effective than SAP therapy. Research has indicated that closed-loop systems lead to improved control of average blood glucose, more extended time in range, and fewer side effects, including hypoglycemia ^[^33,34^]^ Additionally, in very young infants with type 1 diabetes, the hybrid closed-loop device significantly improved GC without increasing hypoglycemia occurrences^[^24^]^. The automated insulin delivery (AID) systems’ switch to calibration-free sensors with remote monitoring also improves user experience, which lowers the cost of diabetes care, improves quality of life, and increases device satisfaction^[^35^]^. Closed-loop systems have been shown to improve overall and diabetes-related quality of life by reducing mean glucose levels, improving GC, and reducing time spent above range^[^28^]^. AI and machine learning have recently been included in closed-loop and sensor-augmented insulin delivery systems to improve system performance ^[^36,37^]^ For instance, when compared to more conventional systems like SAP with predictive low glucose management, the advanced hybrid closed-loop system showed better GC, reduced diabetes discomfort, and improved quality of life^[^38^]^.

These technologies are demonstrated in several commercially available systems. In a real-world multicenter study, the MiniMed 780G’s SmartGuard™ algorithm increased mean TIR from roughly 63 to 82% over 6 months without increasing severe hypoglycemia or ketoacidosis^[^30^]^. GC in children with type 1 diabetes aged 2–6 years was significantly improved by the t:slim X2™ pump with Control-IQ® technology (Tandem Diabetes Care, San Diego, USA). Additionally, the first tubeless HCL platform, the Omnipod 5® system (Insulet Corporation, Acton, USA), has demonstrated positive real-world glycemic outcomes in both adults and children^[^39,40^]^.

### Global perspective and access to technology

Disparities in access to advanced diabetes management technologies, such as CGM and insulin pump therapy, are prevalent across different regions and socioeconomic groups. These disparities can perpetuate or widen inequities in health outcomes for individuals with diabetes, particularly for racial and ethnic minorities and those with fewer economic resources. Financial barriers are a primary concern for those with lower educational attainment, as advanced technologies like CGMs, insulin pumps, and closed-loop systems can be costly^[^41,42^]^. Individuals from lower socioeconomic backgrounds often struggle to afford these technologies due to a lack of insurance coverage, high out-of-pocket costs, or limited access to healthcare resources. Cost-related considerations emerge as a critical concern among older individuals with diabetes, with 18% indicating that factors such as cost and insurance coverage act as barriers to their adoption of technology^[^43^]^.

Research indicates that there are significant racial and ethnic disparities in the prescription rates of insulin pumps and CGMs among children, with non-Hispanic Black and Hispanic children being less likely to receive these technologies compared to non-Hispanic White children, regardless of socioeconomic status^[^44,45^]^. Similarly, these disparities persist among adults with type 1 diabetes, where non-Hispanic White individuals are notably more likely to be prescribed diabetes-related technologies compared to other racial and ethnic groups, and these differences endure even after a 2-year follow-up period^[^46^]^.

Cooperative initiatives and partnerships are essential for tackling the global health challenges linked to diabetes care. These collaborative efforts bring together a variety of stakeholders, including healthcare systems, community organizations, and interdisciplinary teams. Their collective aim is to enhance diabetes management strategies and improve overall outcomes. Efforts such as Project ECHO aim to address global challenges in diabetes care by establishing virtual communities, enhancing knowledge sharing, improving patient outcomes, and implementing quality improvement practices within primary care settings.^[^47^]^.

Before human trials, preclinical research in both small and large animal models is still necessary to test new insulin delivery methods (bioelectronic patches, implantable pumps, and innovative infusion routes) and control algorithms. Large animals (pigs) are commonly used for surgical device prototyping due to their anatomical similarities to humans, and canine and rodent models have been used to test implantable infusion systems and closed-loop controllers, as well as to characterize insulin–glucose dynamics. The translational safety and performance of devices are directly impacted by the pharmacokinetics, device biocompatibility, infusion reliability, and surgical implantation techniques that are informed by these models^[^48,49^]^. By enabling accurate species-specific anatomical models and the virtual rehearsal of implant procedures, advances in AI in anatomical modeling, 3D reconstruction, and educational simulation improve preclinical work. AI can also help with the automated segmentation of imaging data for device design. The potential and limitations of AI in animal anatomy and veterinary education are discussed in recent papers, along with ethical and accuracy issues. Before using AI outputs in device development or training, it is advised to validate them carefully against expert anatomical knowledge. Prototype design may be accelerated by incorporating AI tools into preliminary workflows, but developers must consider model bias, the limited availability of annotated datasets, and the need for human oversight^[^50,51^]^.

## Limitations and future directions

### Current challenges and barriers

Progress in sensor-augmented and closed-loop insulin delivery systems for diabetes care is moving toward fully automated operation, eliminating the need for user input. These advancements also include incorporating glucagon to reduce the risk of hypoglycemia and improve the rapidity of insulin action onset and offset.^[^10^]^

The process of completely optimizing closed-loop systems is still in progress. Their broad use is nevertheless hampered by drawbacks such as slow glucose monitoring, the necessity for frequent recalibration, high costs, and complicated technological issues. The limitations also include healthcare providers being slow to change, limited resources to adopt new technology, difficulties managing insulin levels during exercise, discomfort from wearing devices, doubts about device effectiveness, high costs, inadequate healthcare support, unequal access to technology, and psychological barriers. Overcoming these hurdles is crucial for maximizing the health benefits and ensuring fair access to advanced diabetes management technologies for adults and children with type 1 diabetes^[^51^]^.

### Future innovations

The next phase of closed-loop therapy is anticipated to combine advancements in sensor technology, faster-acting insulins or alternative delivery methods, and advanced algorithms. These innovations aim not only to account for individuals’ diverse responses to glucose management but also to adapt to the daily variations in glucose regulation^[^10^]^. AI is becoming more widely acknowledged as a revolutionary supplement to insulin delivery methods, augmenting sensor-augmented and closed-loop systems. The potential and difficulties of integrating AI into clinical operations are best illustrated by large language models like ChatGPT. AI-driven platforms could help with customized insulin titration, predict hypoglycemia episodes, and improve closed-loop algorithms in the context of diabetes treatment by learning from massive patient behavior and physiology datasets. However, it is important to recognize constraints like algorithm transparency, cybersecurity flaws, and unequal access in low-resource environments^[^52^]^.

### Contraindications and safety considerations

Relative contraindications include severe mental illness, insufficient access to supplies, lack of appropriate local clinical support, and incapacity or unwillingness to self-manage (frequent glucose checks, infusion-set changes). Absolute contraindications to continuing insulin-pump therapy in the hospital or acute setting are usually diabetic ketoacidosis or impaired consciousness. To manage both regular treatment and infrequent device failures, consensus guidelines emphasize cautious patient selection, training, and the need for continuous clinical support^[^6^]^.

## Conclusion

The development of closed-loop insulin administration devices is a significant advancement in the management of diabetes. These systems automate and optimize GC by combining insulin pumps, CGM, and advanced control algorithms. Closed-loop systems are more effective than conventional techniques, such as CSII and SAP therapy, at reducing hypoglycemia, increasing TIR glucose levels, and easing the burden of self-management for people with type 1 and type 2 diabetes. Both elderly persons with long-term type 1 diabetes and adolescents with poorly managed diabetes have found the technology to be safe and effective. Hence, it shows remarkable effectiveness across a variety of demographics, from adolescents to older adults, and even in real-world, unsupervised settings. Dual-hormone systems further enhance glucose control but come with added complexity. As the science develops further, the artificial pancreas continues to hold out hope for enhancing the long-term health and quality of life for millions of people with diabetes.

## Data Availability

All data used in this narrative review are publicly available and sourced from previously published studies. No new data were generated for this work. All included articles have been appropriately cited within the manuscript and are available through the references section.

## Research registration unique identifying number (UIN)

The research registration unique identifying number (UIN) was not required because it is a narrative review.
